# Utility of passive photography to objectively audit built environment features of active transport journeys: an observational study

**DOI:** 10.1186/1476-072X-12-20

**Published:** 2013-04-10

**Authors:** Melody Oliver, Aiden R Doherty, Paul Kelly, Hannah M Badland, Suzanne Mavoa, Janine Shepherd, Jacqueline Kerr, Simon Marshall, Alexander Hamilton, Charlie Foster

**Affiliations:** 1 Human Potential Centre, National Institute for Public Health and Mental Health Research, Auckland University of Technology, Auckland, New Zealand; 2 British Heart Foundation Health Promotion Research Group, Department of Public Health, University of Oxford, Oxford, United Kingdom; 3 McCaughey VicHealth Centre for the Promotion of Mental Health and Community Wellbeing, the University of Melbourne, Melbourne, Australia; 4 San Diego State University, San Diego, USA; 5 CLARITY: Centre for Sensor Web Technologies, Dublin City University, Dublin, Ireland

**Keywords:** Walking, Cycling, SenseCam, Measure, Physical activity

## Abstract

**Background:**

Active transport can contribute to physical activity accumulation and improved health in adults. The built environment is an established associate of active transport behaviours; however, assessment of environmental features encountered during journeys remains challenging. The purpose of this study was to examine the utility of wearable cameras to objectively audit and quantify environmental features along work-related walking and cycling routes.

**Methods:**

A convenience sample of employed adults was recruited in New Zealand, in June 2011. Participants wore a SenseCam for all journeys over three weekdays and completed travel diaries and demographic questionnaires. SenseCam images for work-related active transport journeys were coded for presence of environmental features hypothesised to be related to active transport. Differences in presence of features by transport mode and in participant-reported and SenseCam-derived journey duration were determined using two-sample tests of proportion and an independent samples t-test, respectively.

**Results:**

Fifteen adults participated in the study, yielding 1749 SenseCam images from 30 work-related active transport journeys for coding. Significant differences in presence of features were found between walking and cycling journeys. Almost a quarter of images were uncodeable due to being too dark to determine features. There was a non-significant tendency for respondents to under-report their journey duration.

**Conclusion:**

This study provides proof of concept for the use of the SenseCam to capture built environment data in real time that may be related to active transportation. Further work is required to test and refine coding methodologies across a range of settings, travel behaviours, and demographic groups.

## Introduction

Active transport (e.g., walking or cycling for travel) has been shown to reduce the risk of obesity, hypertension, type 2 diabetes, cardiovascular disease, and overall mortality [1-6]. Active transport provides the opportunity to regularly engage in physical activity that is integrated into daily routines [1,7], and can potentially overcome time constraints, a commonly cited barrier to physical activity engagement in adults [8]. A body of research shows associations between active transport and built environment characteristics (e.g., presence of sidewalks/footpaths, mixed use, safety features, etc.) [9-11], with time and travel distances facilitating or inhibiting the behaviour [1,12,13].

Much of the research to date has utilised self-reported measures of active transport journeys (e.g., travel diaries) rather than objective measures of travel and/or the environment [9]. Inaccuracies of self-reported travel behaviours have been well documented [14,15]. Recent technological advancements, such as accelerometers, global positioning systems (GPS) units, and geographic information systems (GIS), show promise for objectively assessing the geographical location and characteristics of physical activity participation [16]. Moreover, photography through wearable camera technologies (e.g., SenseCam) is increasingly being seen as valuable for eliciting environmental information from the participants’ perspective [17]. This method reduces reliance on participant recall and reporting of environmental features, and facilitates the detailed description of environmental factors, including aesthetics, context, and quality. The capture of such information from a first person point of view at the time of exposure (e.g., the ‘greenness’ of walking environments, traffic volumes, temporary obstructions to cycling) cannot be replicated by current alternative objective methods [18].

Manually coding and classifying these environmental features is prohibitively time consuming and costly to implement, even in small-scale research (e.g., <10 participants). Accordingly, new methodologies are being developed to automate treatment of the photographic data [19]. In order for processes to be automated, machine-learning algorithms need to be developed that can correctly classify images that have been manually “ground-truthed” (i.e., using direct observation or expert opinion). The algorithm can then be used to code images in the absence of human verification. The current study details findings from this first stage of research, by examining a proof of concept for the utility of SenseCam-derived photographic data to enable the objective quantification of built environment features along work-related walking and cycling routes (active transport journeys). Data can be used to develop a classification system for environmental features captured using passive photography that may be important for classifying environmental features of active travel and encouraging or discouraging active transportation behaviours.

## Methods

### Protocol and measures

This was an observational study conducted in Auckland, New Zealand, in June 2011 (winter). Convenience sampling was utilised to invite fifteen adult employees from two universities to participate in the study. Consenting participants were provided with a SenseCam device and requested to wear the unit on a lanyard around their neck for all journeys over three weekdays. They were also asked to record information about their journeys over this time using a 3 day travel diary based on the United Kingdom Department for Transport National Travel Survey [20]. Demographic information was collected via pen-and-paper questionnaire. Ethical approval was provided by the Host Institution ethics committee (AUTEC 11/114, May 25th 2011).

The SenseCam is a small (6 × 7 × 1.5 cm), lightweight (approx 175g), wearable camera, fitted with a wide-angle (fish-eye) lens [21]. When worn on a lanyard around the neck, first-person point-of-view images from the wearer are captured. The SenseCam contains an internal clock, tri-axial accelerometer, magnetometer, light-intensity and light-colour sensors, a passive infrared (body heat) detector, and a temperature sensor. The camera automatically takes images when triggered by changes in the sensor information collected [21]. On average, this results in image capture every 10 seconds during travel behaviour [22]. When not triggered by sensor data, images are automatically captured every 50 seconds. Images can also be manually captured by the wearer, but not viewed as there is no viewfinder.

Potential environmental features that may be captured from SenseCam images were defined a-priori from a suite of commonly used environmental audits in active transport research as follows: Neighborhood Walking Environment Scale (NEWS) [23]; Systematic Pedestrian and Cycling Environmental Scale (SPACES) [12]; and the Walking and Bicycling Suitability Assessment Forms (WABSA-W and WABSA-B, respectively) [24]. Additional features that were identified as potential influences of active transport that are not captured in these audits were also identified and included (e.g., weather and temporary obstructions to walking or cycling). In total, 30 common active transport-related environmental features were identified that may potentially be identified from SenseCam images (Table 1).

**Table 1:** Description of environmental features present in walking and cycling journeys

**Feature**	**Description**	**Cycling (n = 599**^**†**^**)**		**Walking (n = 1150**^**†**^**)**		**Total (n = 1749**^**†**^**)**	
		**n**	**(%)**	**n**	**(%)**	**n**	**(%)**
Bus stop	Bus stop visible in photo	44	7.3	69	6.0	113	6.5
Cars driving	Cars in motion or in traffic lanes on road	388	64.8	674	58.6*	1062	60.7
Cars in carpark	Cars parked in car park wholly or more than 2/3 partially visible	68	11.4	110	9.6	178	10.2
Cars parked	Cars parked on side of the road	190	31.7	151	13.1**	341	19.5
Commercial	Commercial or institutional buildings visible	281	46.9	648	56.3**	929	53.1
Congested traffic	More than 6 stationary cars in driving lanes	4	0.7	10	0.9	14	0.8
Cycle lanes	Designated cycle lane on road or footpath	16	2.7	247	21.5**	263	15.0
Cyclists	Any person/people riding cycles other than the participant	6	1.0	8	0.7	14	0.8
Dark	Image indicates journey conducted in darkness (e.g., dusk or dawn, streetlights on) but features still visible and image codeable^†^	120	20.0	209	18.2	329	18.8
Dogs	Dogs or a lead in participant hand visible	0	0.0	4	0.3	4	0.2
Footpath	Footpath visible (not walkway/pathway)	338	56.4	761	66.2**	1099	62.8
Footpath good condition	No cracks or potholes visible	327	54.6	759	66.0**	1086	62.1
Graffiti	Graffiti visible	0	0.0	2	0.2	2	0.1
Grass verge	Any area of grass either beside road or footpath	270	45.1	504	43.8	774	44.3
Grass verge maintained	No obvious weeds or overgrown grass	262	43.7	454	39.5	716	40.9
Litter	Litter present (e.g., paper, food wrappings, etc.)	1	0.2	1	0.1	2	0.1
Other lights	Lights from houses, buildings or cars in photos	247	41.2	348	30.3**	595	34.0
Pedestrian crossing	Zebra crossings and traffic light pedestrian crossings visible	82	13.7	240	20.9**	322	18.4
Pedestrians	Any person/people in the photo other than the participant	63	10.5	272	23.7**	335	19.2
Permanent obstructions to cycling	Tree, signage, or other permanent structure in cycleway	2	0.3	0	0.0	2	0.1
Permanent obstructions to walking	Tree, signage, or other permanent structure on footpath/walkway	2	0.3	0	0.0	2	0.1
Rain	Rain visible	63	10.5	54	4.7**	117	6.7
Residential	Private homes visible	155	25.9	229	19.9**	384	22.0
Retail buildings	Buildings with retail/shop-fronts visible	141	23.5	165	14.3**	306	17.5
Road good condition	No cracks or potholes visible	462	77.1	820	71.3**	1282	73.3
Street lighting	Street lights visible (not including traffic lights)	209	34.9	531	46.2**	740	42.3
Temporary obstructions to cycling	Rubbish bins, parked cars, roadworks, etc. in cycleways	9	1.5	11	1.0	20	1.1
Temporary obstructions to walking	Rubbish bins, parked cars, roadworks, etc. on footpath/walkway	14	2.3	41	3.6	55	3.1
Trees	Any trees visible in photo including from a distance	441	73.6	842	73.2	1283	73.4
Walkway	Journey occurring in walkway/pathway (not road or footpath)	45	7.5	200	17.4**	245	14.0

Notes: Data were collected in Auckland, New Zealand, in June 2011.

n = number of images.

*p < 0.05; **p < 0.01 significant difference in features present between walking and cycling journeys; ^†^If photo was too dark to code individual features then it was coded as uncodeable and not included here; %, percentage of walking or cycling images where feature was present; n, number of images.

Numerous privacy and ethical issues exist when using Passive Automated Digital Image-capture (PADI) devices such as the SenseCam. Considerations include passive versus purposive data collection, intrusiveness, informed consent, privacy issues (e.g., data collection in sensitive situations), mitigation of loss of confidentiality, and data collection from unconsenting third parties. The protocols and procedures of the current study adhered to the ethical framework proposed by Kelly et al. for use of PADI devices in research [25].

### Data treatment

Descriptive information for participant demographics was calculated. Body Mass Index (BMI) was determined from self-reported height and weight as follows: weight in kilograms/height in meters^2^. World Health Organization thresholds for overweight and obesity were then applied (≥25 kg/m^2^ and ≥30 kg/m^2^, respectively) [26]; SenseCam data were downloaded into the Oxford CLARITY SenseCam Browser software (freely available at http://sensecambrowser.codeplex.com). Participants were shown how to browse images and were provided the opportunity to review and delete any images in private if they wished to do so. This SenseCam Browser software automatically groups images into a series of distinct events utilising the accelerometer data [27]. Active transport journeys to or from workplace were identified using a combination of participant-reported journeys derived from the travel diaries, SenseCam-derived events, and manual scanning of SenseCam images. Focusing on journeys between home and workplace only was a pragmatic decision to ensure manageability of data treatment and acknowledging the significant contribution work-related travel makes to overall travel behaviours. Travel diaries were considered the criterion for occurrence and mode of trips undertaken. Although GPS data can be used to identify walking and cycling journeys, trip purpose is not captured, therefore the travel diary was deemed an appropriate measure of work-related journey occurrence for the purposes of the current study. SenseCam images were considered the criterion for journey start and end times, which were determined using the protocol of Kelly et al. [22] and active transport events (journeys) for walking or cycling set accordingly. Where trip-chaining occurred (identified from the SenseCam images as stopping at one or more locations between home and work (e.g., shops or cafe), separate events were set for the active transport components of these journeys. Descriptive data (e.g., image filename, date, time) was then extracted as a .csv file for walking or cycling journeys between home and work only using the SQL database manager (http://sensecambrowser.codeplex.com/documentation), and saved in Microsoft Excel 2007 (Microsoft Corp, Redmond, WA).

Individual trip-chain events were collated into their respective active transport journeys. Participant-reported journeys of less than 10 minutes duration were excluded from analyses. Evidence suggests that a minimum bout of 10 minutes of self-reported physical activity is required for health benefits [28,29]. Delimiting data treatment to journeys of at least 10 minutes duration was thus a practical approach to maintaining data treatment manageability and ensuring that data retained were relevant to the field of physical activity and health research. Active transport components of mixed mode journeys (e.g., parking the car and walking) were only retained where they met the 10 minute data inclusion threshold. Individual images were then scanned for each feature listed in Table 1 and given a binary code of 1 or 0 to indicate whether the feature was present or not, respectively. Inter-rater reliability was calculated for 10% of randomly selected images using Cohen’s kappa (κ) statistic [30]. Descriptive data were calculated for frequency of features present by travel mode. To examine content validity, differences in absence or presence of features by transport mode were determined using two-sample tests of proportion. Differences in participant-reported and SenseCam-derived journey duration were calculated using an independent samples t-test. Analyses were undertaken using STATA IC 10.1 (StataCorp, TX). Statistical significance was set at α = 0.05.

## Results

**Figure 1:**
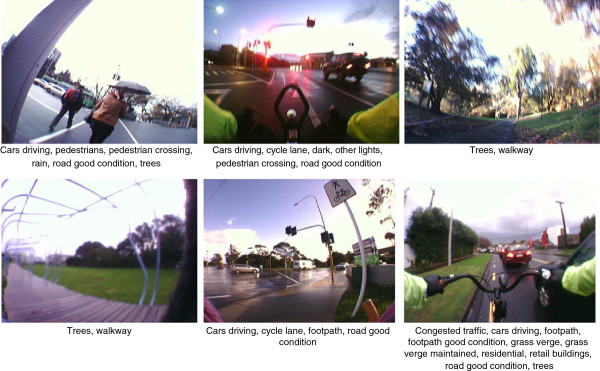
**Sample images and exemplar coding of features present.** Note: Data were collected in Auckland, New Zealand, in June 2011.

All fifteen participants provided informed consent to participate in the study. Table 2 shows the descriptive information for participant characteristics. One participant did not complete his travel diary, five reported no active transport journeys, and one reported one non work-related active journey. A total of 44 work-related active transport journeys were captured from the remaining eight participants over the three days. Three trips were removed from further analysis; two because they were reported to be less than 10 minutes duration, and one because there was no corresponding SenseCam data for the reported journey in the travel diary (the SenseCam was delivered after the journey was reported). There were 10 instances of trip-chaining, involving 21 separate events (one trip-chain comprised three events). After collating trip-chains into individual journeys, the remaining sample included 30 work-related active transport journeys (yielding 2292 images in total). Almost a quarter (23.7%, n = 543) of images were uncodeable due to being too dark to determine features, a proportion that was relatively similar for walking and cycling trips (23.1% and 24.8% uncodeable, respectively). In total, 1749 photos were coded for environmental features. An example of images captured and corresponding coding is provided in Figure 1.

**Table 2:** Participant characteristics (n (%) unless stated otherwise)

**Variable**	**n**	**(%)**
Age (mean (SD))	38.6	(10.7)
Sex		
Male	3	(20.0)
Female	12	(80.0)
BMI		
Normal/Underweight (<25 kg/m^2^)	9	(60.0)
Overweight (≥25 kg/m^2^)	6	(40.0)
Obese (≥30 kg/m^2^)	0	(0.0)
Occupation		
Professor	1	(6.7)
Senior Lecturer	1	(6.7)
Researcher	7	(46.7)
Research administrator/assistant	4	(26.7)
Teaching assistant	1	(6.7)
Technician	1	(6.7)
Site		
Central city	3	(20.0)
Suburban	12	(80.0)

Notes: Data were collected in Auckland, New Zealand, in June 2011.

BMI, Body Mass Index (kg/m^2^); n, number of participants; SD, Standard Deviation.

Significant differences in presence of features were found between walking and cycling journeys, in directions that would be expected (e.g., greater proportion of footpaths and pedestrians found for walking journeys compared with cycling journeys; Table 1). Average active transport journey duration was 21.7 minutes and there was a non-significant tendency for respondents to under-report their journey duration, which was greater in cyclists than walkers (Table 3). Inter-rater reliability for presence or quality of features was considered acceptable (range κ = 0.56-0.95 across all features [31]).

**Table 3:** Journey duration characteristics

**Trip duration (minutes)**	**Cycling (n = 8)**		**Walking (n = 21)**		**Total (n = 29)**	
	**Mean**	**(min, max)**	**Mean**	**(min, max)**	**Mean**	**(min, max)**
Reported duration	20.1	(15.0, 53.0)	22.0	(10.0, 45.0)	21.5	(10.0, 53.0)
SenseCam duration	21.3	(9.9, 56.6)	22.3	(9.6, 60.0)	21.7	(9.6, 56.6)
Difference (Reported – SenseCam)	−1.1	(−3.6, 5.2)	−0.2	(−11.1, 8.6)	−0.42	(−11.1, 8.6)

Notes: Data were collected in Auckland, New Zealand, in June 2011.

n = number of journeys.

One walking journey was extracted for the comparison between reported and SenseCam trip duration as this was not recorded on the travel diary and would have biased the comparisons.

## Discussion

This study provides support for the utility of the SenseCam to capture contextual features within the built environment that individuals may encounter during walking or cycling journeys. Although the current dataset was derived from a limited number of participants and geographic area, all hypothesised features of importance identified from the audit tools were identified from the images captured. The tendency to under-report journey duration is in contrast with previous SenseCam research [22,32] and Global Positioning System (GPS) studies [33], possibly due the small convenience sample and focus on work related walking and cycling trips only. We found significant differences in the presence of specific features between walking and cycling modes, suggesting preliminary support for the content validity of this approach. For example, a significantly greater proportion of footpaths, pedestrians, and pedestrian crossings were found for walking trips, while a higher prevalence of car presence was found for cycling journeys. With the exception of cycle lanes, all significant differences between features identified by walking and cycling were in the expected direction. The lack of cycle lanes in the study areas may explain this finding somewhat, whereby many cycling journeys were completed on roads without cycle lanes. Improving on existing audits that do not reflect temporal exposure, use of the SenseCam data enabled the capture of factors that individuals actually encountered during active transport journeys, such as traffic density; weather conditions; presence of pedestrians, cyclists, and dogs; and temporary obstructions to walking or cycling.

Almost a quarter of data were lost due to images being too dark to enable coding of features. In part, this is likely due to the study being conducted during winter, with reduced daylight hours and work-related travel occurring in reduced light. In some instances participants may have intentionally or unintentionally worn the SenseCam with the lens facing inwards or worn an item of clothing over the SenseCam, which would also result in uncodeable images. Researchers may benefit from asking participants whether there were instances where the SenseCam lens was intentionally obscured to account for this. A high proportion of work-related journeys were omitted due to the use of motorised transport modes. Again, this may be due to the study being conducted during winter with weather conditions discouraging active transport modes. It is also likely that some participants resided in environments that were unsupportive for actively transporting to their workplaces, however we cannot establish this from the current investigation.

While employment of photography in health research is not a new concept, the use of wearable cameras to passively capture a series of images over specified times has only been possible in recent years. As we have observed with other emerging technologies in health behaviour research such as pedometry, accelerometry, and GPS, there is a significant amount of research that is first required to develop appropriate and agreed-upon data treatment methods. As noted earlier, this study was conducted with a small sample and was limited to two areas of Auckland, New Zealand, only. Our aim was not to provide a comprehensive framework for coding environmental features, but to provide proof of concept and baseline data for future active transport work across international sites. Research is now needed to determine criterion and predictive validity of SenseCam image coding of environmental features over a range of settings and situations (e.g., heavy traffic) and utilising the wide range of validated environmental audits available. SenseCam images can provide repeated measures of environmental variables encountered during journeys, which may differ by individual, and by journey duration, purpose, and mode. As such it is possible that some journeys or individuals may bias findings (e.g., due to having a greater number of repeated measures of one factor). Future research should thus consider accounting for clustering of environmental features both within journeys and individuals when investigating differences between environmental features encountered. Manual coding of the data was time consuming, taking approximately 25 researcher hours to process the 2292 images (equivalent to approximately 6.4 hours of journey time). Consequently, automated concept detection techniques need to be extended to identify environmental features of interest in future research with larger sample sizes [19]. The wide range of kappa values found for inter-rater reliability across factors may denote issues with researcher interpretation (such as features being in ‘good condition’) or difficulties in clearly establishing features (due to photos being blurry for example). Future work to establish clear coding instructions and training protocols for researchers is thus required. Further research is also needed to consider more detailed built environment features than those presented here, for example types of pedestrian crossings, which may be especially important for vulnerable populations. Walking and cycling were the only travel modes examined in the current study; future research should consider the implications of differing travel modes and travel behaviours on image quality (e.g., running may result in blurry/uncodeable images), across a wider range of journey purposes and demographic groups.

## Conclusions

This study provides proof of concept for the use of the SenseCam to capture data on built environment features that may be related to active transportation. Having these temporal contextual data to support environmental audits or GIS-derived built environment measures may improve sensitivity of measures and improve our ability to establish exposures and explain individual preferences for transport mode and routes taken. Considerable opportunities exist to harness the in-depth contextual information on built environments that are captured by SenseCam. This study provides the first step towards understanding these opportunities in relation to active transport.

## Abbreviations

BMI: Body mass index; NEWS: Neighborhood walking environment scale; PADI: Passive automated digital image-capture; SPACES: Systematic pedestrian and cycling environmental scale; WABSA-B: Bicycling suitability assessment form; WABSA-B: Walking suitability assessment form.

## Competing interest

The authors have no conflicts of interest to declare.

## Authors’ contributions

MO, ARD, PK, HMB, and SM conceived and undertook the study. JS carried out the SenseCam data treatment. MO drafted the manuscript and performed the statistical analysis. JK, SM, AH, and CF participated in the design of the study. All authors contributed to drafting the manuscript, and have read and approved the final manuscript.

## References

[B1] Badland H Schofield G Garrett N Travel behavior and objectively measured urban design variables: associations for adults traveling to work Health Place 2008 14 85 95 10.1016/j.healthplace.2007.05.002 17590378

[B2] Hayashi T Tsumura K Suematsu C Okada K Fuji S Endo G Walking to work and the risk for hypertension in men: the Osaka health survey Ann Intern Med 1999 130 21 26 10391811 10.7326/0003-4819-131-1-199907060-00005

[B3] Hu G Pekkarinen H Hanninen O Tian H Guo Z Relation between commuting, leisure time physical activity and serum lipids in a Chinese urban population Ann Hum Biol 2001 28 412 421 10.1080/03014460010016671 11459239

[B4] Hu G Pekkarinen H Hanninen O Yu Z Guo Z Tian H Commuting, leisure-time physical activity, and cardiovascular risk factors in China Med Sci Sports Exerc 2002 34 234 238 10.1097/00005768-200202000-00009 11828231

[B5] Hu FB Li TY Colditz GA Willett WC Manson JE Television watching and other sedentary behaviors in relation to risk of obesity and type 2 diabetes mellitus in women J Am Med Assoc 2003 289 1785 1791 10.1001/jama.289.14.1785 12684356

[B6] Hamer M Chida Y Active commuting and cardiovascular risk: a meta-analytic review Prev Med 2008 46 9 13 10.1016/j.ypmed.2007.03.006 17475317

[B7] Bull FC Gauvin L Bauman A Shilton T Kohl HW Salmon A The Toronto charter for physical activity: a global call for action J Phys Act Health 2010 7 421 422 20683082 10.1123/jpah.7.4.421

[B8] Sullivan C Oakden J Young J Butcher H Lawson R Obstacles to action: A study of New Zealanders' physical activity and nutrition 2003 Wellington, New Zealand: Sport and Recreation New Zealand

[B9] Badland H Schofield G Transport, urban design, and physical activity: an evidence-based update Transportation Res 2005 Part D 177 196

[B10] Transportation Research Board and Institute of Medicine of the National Academies Does the Built Environment Influence Physical Activity? Examining the Evidence 2005 Washington, DC: Transportation Research Board, Institute of Medicine of the National Academies

[B11] Saelens BE Sallis JF Frank LD Environmental correlates of walking and cycling: findings from the transportation, urban design, and planning literatures Ann Behav Med 2003 25 80 91 10.1207/S15324796ABM2502_03 12704009

[B12] Pikora TJ Giles-Corti B Kkuiman MW Bull FC Jamrozik K Donovan RJ Neighborhood environmental factors correlated with walking near home: using SPACES Med Sci Sports Exerc 2006 38 708 718 10.1249/01.mss.0000210189.64458.f3 16679987

[B13] Duncan M Mummery W GIS or GPS? A comparison of the two methods for assessing route taken during active transport Am J Prev Med 2007 33 51 53 10.1016/j.amepre.2007.02.042 17572312

[B14] Golob TT Meurs H Biases in response over time in a seven-day travel diary Transportation 1986 13 163 181 10.1007/BF00165546

[B15] Clarke M Dix M Jones P Error and uncertainty in travel surveys Transportation 1981 10 105 126 10.1007/BF00165261

[B16] Oliver M Badland HM Mavoa S Duncan MJ Duncan JS Combining GPS, GIS and accelerometry: methodological issues in the assessment of location and intensity of travel behaviors J Phys Act Health 2010 7 102 108 20231761 10.1123/jpah.7.1.102

[B17] Wang C Burris MA Photovoice: concept, methodology, and use for participatory needs assessment Health Educ Behav 1997 24 369 387 10.1177/109019819702400309 9158980

[B18] Weber K Mobile Devices and a New Understanding of Presence 2010 Copenhagen, Denmark: Falko Schmid, Tobias Hesselmann, Susanne Boll, Keith Cheverst, Lars Kulik (Chairs)

[B19] Doherty AR Caprani N Conaire CÓ Kalnikaite V Gurrin C Smeaton AF O’Connor NE Passively recognising human activities through lifelogging Comput Hum Behav 2011 27 1948 1958 10.1016/j.chb.2011.05.002

[B20] Department for Transport National Travel Survey 2009: Statistical release 2010 London: Author

[B21] Hodges S Williams L Berry E Izadi S Srinivasan J Butler A Smyth G Kapur N Wood K SenseCam: A Retrospective Memory Aid UbiComp: 8th International Conference on Ubiquitous Computing vol 4602 of LNCS 2006 Berlin, Heidelberg: Springer 177 193

[B22] Kelly P Doherty A Berry E Hodges S Batterham AM Foster C Can we use digital life-log images to investigate active and sedentary travel behaviour? Results from a pilot study Int J Behav Nutr Phys Act 2011 8 44 10.1186/1479-5868-8-44 21599935 PMC3118309

[B23] Cerin E Saelens BE Sallis JF Frank LD Neighborhood environment walkability scale: validity and development of a short form Med Sci Sports Exerc 2006 38 1682 1691 10.1249/01.mss.0000227639.83607.4d 16960531

[B24] Emery J Crump C The WABSA Project: Assessing and Improving Your Community’s Walkability & Bikeability 2003 North Carolina: Department of Health Behavior and Health Education, School of Public Health, The University of North Carolina at Chapel Hill

[B25] Kelly P Marshall S Badland H Kerr J Oliver M Doherty AR Foster C Ethics of using Passive Automated Digital Image-capture (PADI) devices in health behaviour research Am J Prev Med 2013 44 314 319 10.1016/j.amepre.2012.11.006 23415131

[B26] World Health Organization Obesity: Preventing and Managing the Global Epidemic: Report of a WHO Consultation 2004 Geneva, Switzerland: Author 11234459

[B27] Doherty AR Pauly-Takacs K Caprani N Gurrin C Moulin CJA O'Connor NE Smeaton AF Experiences of aiding autobiographical memory using the SenseCam Human Comput Interact 2012 27 151 174

[B28] Woolf-May K Kearney EM Owen A Jones DW Davison RC Bird SR The efficacy of accumulated short bouts versus single daily bouts of brisk walking in improving aerobic fitness and blood lipid profiles Health Educ Res 1999 14 803 815 10.1093/her/14.6.803 10585387

[B29] Murphy MH Hardman AE Training effects of short and long bouts of brisk walking in sedentary women Med Sci Sports Exerc 1998 30 152 157 9475657 10.1097/00005768-199801000-00021

[B30] Cohen JA A coefficient of agreement for nominal scales Educ Psychol Meas 1960 20 37 46 10.1177/001316446002000104

[B31] Landis JR Koch GG The measurement of observer agreement for categorical data Biometrics 1977 33 159 174 10.2307/2529310 843571

[B32] Kelly P Doherty AR Hamilton A Matthews A Batterham AM Nelson M Foster C Cowburn G Evaluating the feasibility of measuring travel to school using a wearable camera Am J Prev Med 2012 43 546 550 10.1016/j.amepre.2012.07.027 23079179 PMC3474949

[B33] Department for Transport National Travel Survey 2011 GPS pilot: Summary analysis 2011 London: Author

